# Setting the pace: host rhythmic behaviour and gene expression patterns in the facultatively symbiotic cnidarian *Aiptasia* are determined largely by *Symbiodinium*

**DOI:** 10.1186/s40168-018-0465-9

**Published:** 2018-05-09

**Authors:** Michal Sorek, Yisrael Schnytzer, Hiba Waldman Ben-Asher, Vered Chalifa Caspi, Chii-Shiarng Chen, David J. Miller, Oren Levy

**Affiliations:** 1 The Mina & Everard Goodman Faculty of Life Sciences, Bar-Ilan University, 52900 Ramat-Gan, Israel; 2 National Institute of Biotechnology in the Negev, Ben-Gurion University of the Negev, Beer-Sheva, Israel; 3 National Museum of Marine Biology and Aquarium, Checheng, Pingtung Taiwan, Republic of China; 4 ARC Centre of Excellence for Coral Reef Studies and Department of Molecular and Cell Biology, James Cook University, Townsville, 4811 Australia

**Keywords:** Symbiotic and aposymbiotic, Sea anemone *Aiptasia diaphana*, Biological clocks, Behaviour, Circadian, Circatidal, Holobiont, Gene expression

## Abstract

**Background:**

All organisms employ biological clocks to anticipate physical changes in the environment; however, the integration of biological clocks in symbiotic systems has received limited attention. In corals, the interpretation of rhythmic behaviours is complicated by the daily oscillations in tissue oxygen tension resulting from the photosynthetic and respiratory activities of the associated algal endosymbiont *Symbiodinium*. In order to better understand the integration of biological clocks in cnidarian hosts of *Symbiodinium*, daily rhythms of behaviour and gene expression were studied in symbiotic and aposymbiotic morphs of the sea-anemone *Aiptasia diaphana*.

**Results:**

The results showed that whereas circatidal (approx. 12-h) cycles of activity and gene expression predominated in aposymbiotic morphs, circadian (approx. 24-h) patterns were the more common in symbiotic morphs, where the expression of a significant number of genes shifted from a 12- to 24-h rhythm. The behavioural experiments on symbiotic *A. diaphana* displayed diel (24-h) rhythmicity in body and tentacle contraction under the light/dark cycles, whereas aposymbiotic morphs showed approximately 12-h (circatidal) rhythmicity. Reinfection experiments represent an important step in understanding the hierarchy of endogenous clocks in symbiotic associations, where the aposymbiotic *Aiptasia* morphs returned to a 24-h behavioural rhythm after repopulation with algae.

**Conclusion:**

Whilst some modification of host metabolism is to be expected, the extent to which the presence of the algae modified host endogenous behavioural and transcriptional rhythms implies that it is the symbionts that influence the pace. Our results clearly demonstrate the importance of the endosymbiotic algae in determining the timing and the duration of the extension and contraction of the body and tentacles and temporal gene expression.

**Electronic supplementary material:**

The online version of this article (10.1186/s40168-018-0465-9) contains supplementary material, which is available to authorized users.

## Background

Coastal ecosystems in general, and coral reefs in particular, are highly diverse and productive environments, but despite the importance of tidal cycles on the resident flora and fauna, the mechanisms by which organisms anticipate and compensate for these daily rhythms remain largely unknown. Whilst an intrinsic circadian clock could potentially generate cycles with tidal periodicity, recent work on a marine crustacean, *Eurydice pulchra,* indicates the presence of an independent circatidal clock [1, 2]. However, the nature of the relationship between the circadian and circatidal clocks (specifically, whether common components are involved or they operate independently), and the question of whether this mechanism is unique to *Eurydice*, is not yet clear. In corals and many other tropical marine invertebrates, the presence of photosynthetic symbionts further complicates the interpretation of rhythmic behaviours, as daily photosynthetic cycles can cause diurnal fluctuations in host tissue oxygenation and nutrient states that then also alter gene expression patterns [3].

The ecological significance of the coral-dinoflagellate symbiosis makes this system particularly important in terms of understanding the integration of biological clocks of two eukaryotes. For coral hosts, this is an obligate relationship [4], thereby ruling out the possibility of studying the regulation of the host clock in the absence of the photosymbiont. However, in symbiotic sea anemones, such as *Aiptasia*, the relationship with *Symbiodinium* is facultative, making this a convenient laboratory surrogate for studying coral symbiosis [5–7].

In order to gain a better understanding of the basis of biological rhythms in cnidarian symbioses, temporal patterns of behaviour and gene expression were investigated in symbiotic and aposymbiotic morphs of the sea anemone *Aiptasia diaphana.* As reported for many other symbiotic cnidarians, the physiology and behaviour of symbiotic *Aiptasia* was predominantly circadian with a 24-h rhythmicity observed for both photosynthesis and tentacle extension/body contraction. In contrast, aposymbiotic morphs displayed circatidal (i.e. approximately 12 h) cycles of tentacle contraction/body extension. Transcriptomics revealed both 12- and 24-h cycles of gene expression but, whereas the majority of genes in aposymbiotic morphs obeyed circatidal rather than diel patterns of expression, the opposite was true of symbiotic anemones. A substantial number (*n* = 227; ~ 10% from 12- and 24-h rhythmicity in symbiotic morph) of host genes shifted from a 12- to a 24-h cycle in the presence of the symbiont (Additional file 1: Table S1). These findings indicate the extent to which the rhythmic gene expression of the cnidarian host is influenced by *Symbiodinium.* The behavioural, physiological, and transcription rhythms of the host were extensively modified in the presence of the symbiotic algae, which therefore appear to represent the “timekeepers” in the association. Interestingly, the observed differences in temporal expression patterns of casein kinase I and peroxiredoxin between aposymbiotic and symbiotic *Aiptasia* morphs, are consistent with the current model for circatidal regulation in the crustacean *Eurydice pulchra* [1, 2]. The results suggest that, as in the case of the circadian clock, mechanisms of circatidal regulation may be conserved across the Metazoa.

## Results

**Fig. 1:**
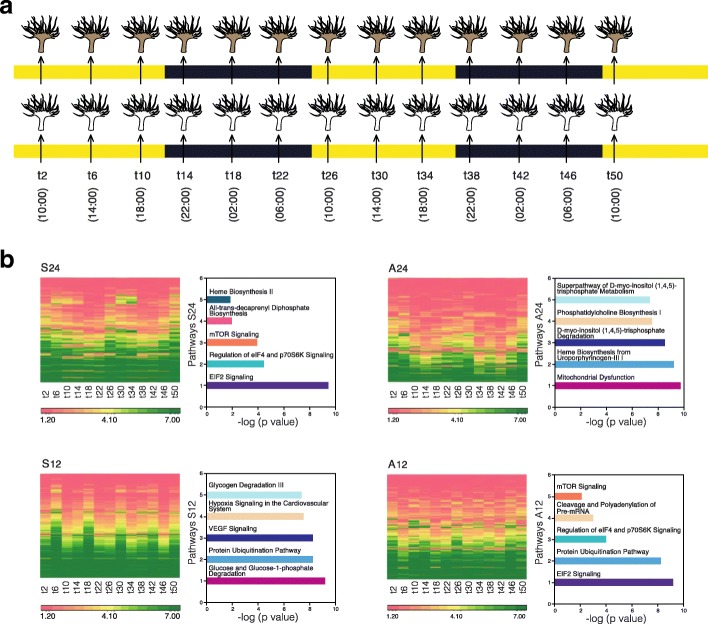
**a** Experimental design—symbiotic and aposymbiotic *Aiptasia* (*N* = 8) were sampled for deep seq analysis over 2 days of LD cycle (12:12). **b** Heat map showing the four groups: S24 (symbiotic morphs with 24-h oscillation), S12 (symbiotic morphs with 12-h oscillation), A24 (aposymbiotic morphs with 24-h oscillation), and A12 (aposymbiotic morphs with 12-h oscillation). The *x* axis indicates time of sampling where *t* indicates the numbers of hours since the lights were turned on at the beginning of the experiment. The five top canonical pathways—comparison between symbiotic and aposymbiotic *Aiptasia* (S24, S12, A24, A12). Analysis of annotated sequences based only on *H. sapiens* annotations was performed using the IPA software. Values for the significance of the pathways are represented as −log (*p* value)

**Fig. 2:**
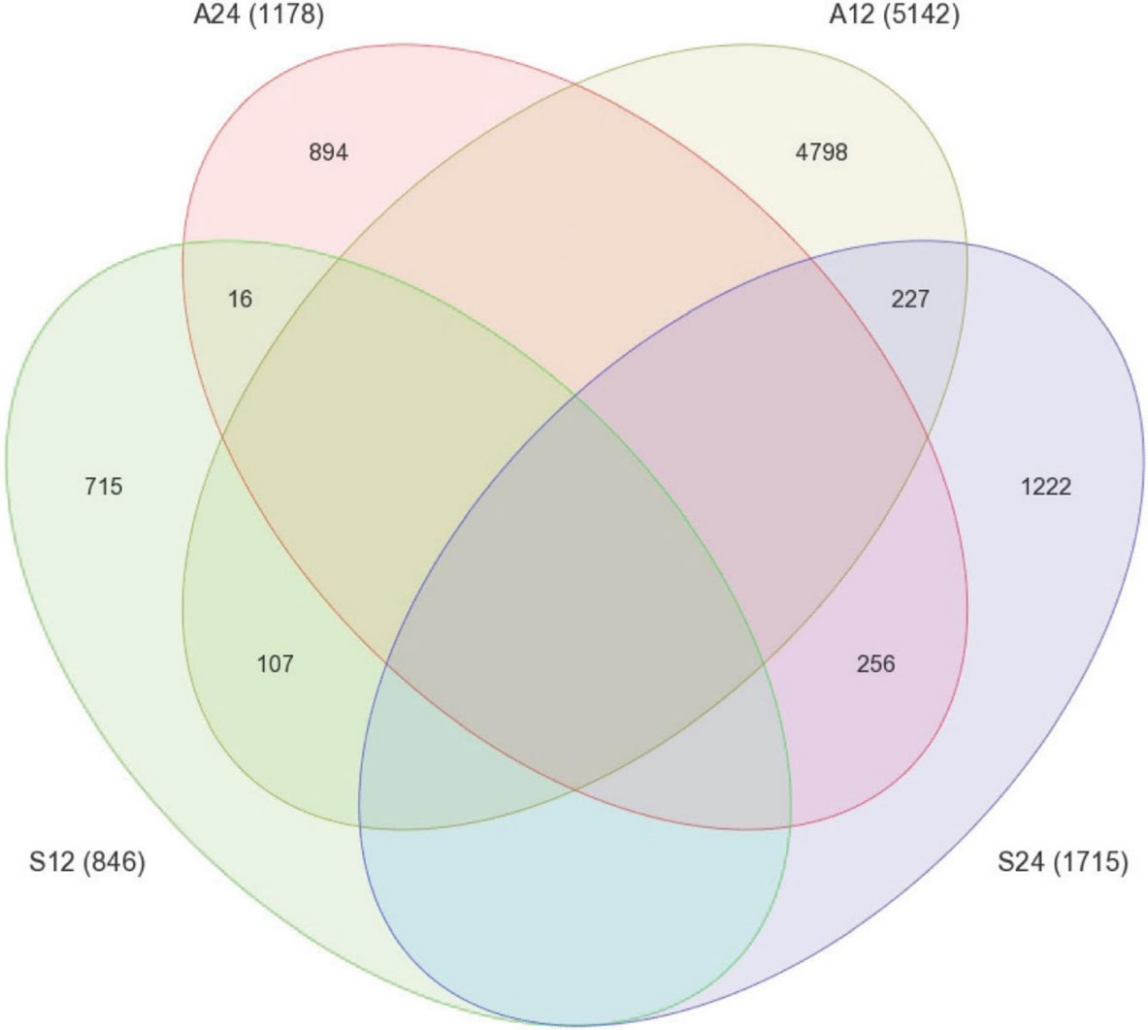
Venn diagram showing the number of constructed contigs in each group, S24, S12, A24, A12, the rhythmicity of each group, and the overlap between them

**Fig. 3:**
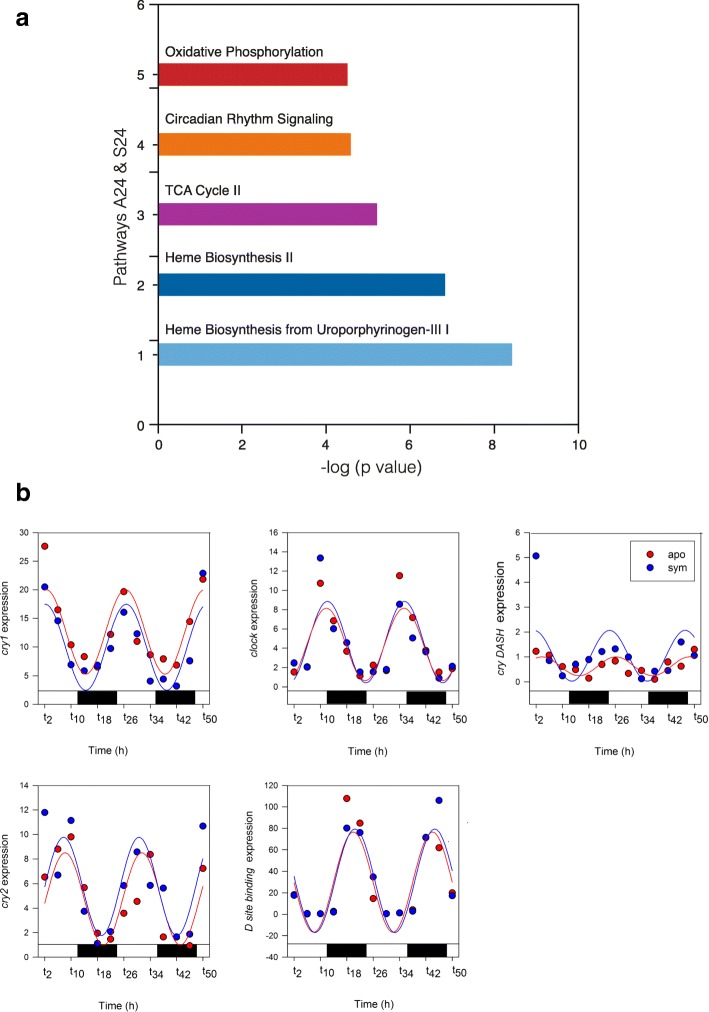
Transcriptome data. **a** The five top canonical pathways—genes common to symbiotic and aposymbiotic *Aiptasia* with rhythmicity of 24 h (S24, A24). Analysis of annotated sequences based only on *H. sapiens* annotations was performed using the IPA software. Values for the significance of the pathways are represented as −log (*p* value). **b** Temporal expression patterns of selected core circadian machinery genes during 2 days of LD (12:12) for symbiotic and aposymbiotic *Aiptasia* with a 24-h rhythm. *P* < 0.05; fitting curve is based on sinusoidal function

**Fig. 4:**
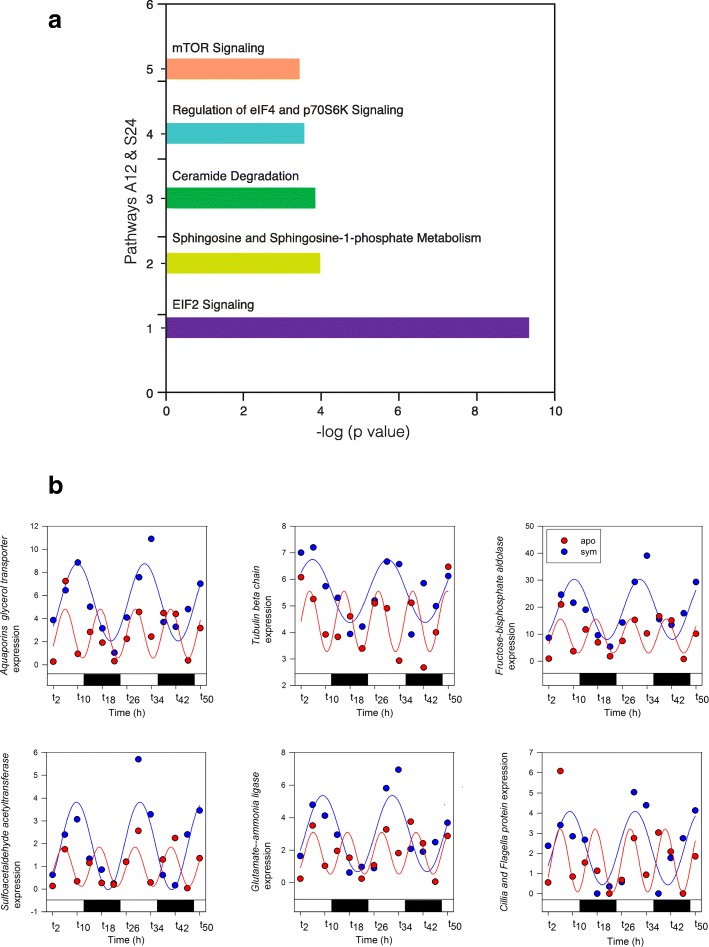
Transcriptome data. **a** The five top canonical pathways genes common to symbiotic and aposymbiotic *Aiptasia* with rhythmicity of 12 or 24 h (S24, A12). Analysis of annotated sequences based only on *H. sapiens* annotations was performed using the IPA software. Values for the significance of the pathways are represented as −log (*p* value). **b** Temporal expression patterns of selected genes during 2 days of LD (12:12) for symbiotic *Aiptasia* with rhythmicity of 24 h and aposymbiotic *Aiptasia* with rhythmicity of 12 h. *P* < 0.05; fitting curve is based on sinusoidal function

Temporal gene expression rhythmicity in *A. diaphana* was investigated by using Illumina RNAseq technology (see the “Methods” section) to follow transcriptomic changes in symbiotic and aposymbiotic samples over 48 h (12-h light/12-h dark cycles; Fig. 1a). The 35,492 contigs that were differentially expressed in the symbiotic and aposymbiotic morphs (Fig. 1b) encoded 44,287 predicted proteins, of which 13,464 were represented in the published protein database for this organism (which contains 29,269 predictions [4]). In order to detect rhythmic expression patterns, the 35,492 contigs were analysed using JTK_Cycle (v2.1) [8–10]. The rhythmic analysis identified 8253 genes (~ 23% of the differentially expressed transcripts) that cycled with either 24- or 12-h periodicity (*p* < 0.05). We could distinguish four patterns amongst these oscillating genes, referred to here as A12 (aposymbionts with 12-h oscillations), A24 (aposymbionts with 24-h oscillations), S12 (symbionts with 12-h oscillations), and S24 (symbionts with 24-h oscillations). Figure 1b summarizes the differential gene expression data identified during the 2 days of light/dark cycling. One striking finding was that, whereas in aposymbiotic *A. diaphana,* the majority of genes cycled with a 12 h (A12 = 5142 genes) rather than 24 h (A24 = 1178) periodicity, the reciprocal was true for symbiotic *A. diaphana,* where more genes cycled with a 24 h (S24 = 1715) than 12 h (S12 = 846) periodicity [see Venn diagram in Fig. 2; RT-PCR validation of the transcriptome analysis is shown in Additional file 2: Figure S1]. Ingenuity Pathway Analysis (IPA) software was used to analyse transcripts for which confident annotation (based on *H. sapiens*) was available. The top five canonical pathways for each group are summarized in Figs. 1b, 3a, and 4a. As in other symbiotic cnidarians [11, 12], the canonical circadian clock components *cry1*, *cry2*, *clock*, and *D site binding protein* displayed diel expression irrespective of the presence of *Symbodinium* (i.e. these are amongst the 256 genes in the A24/S24 group; Fig. 3b); *Cry-dash*, however, exhibited a weaker 24-h rhythm [*p* < 0.04 (apo) and *p* < 0.01(sym)], and a significant (12 h) rhythm for *Timeless* was detected only in aposymbiotic morphs (*p* < 0.002). In contrast, *rhodopsin* was found to display 24-h oscillations only in symbiotic *A. diaphana* (*p* < 0.016).

**Fig. 5:**
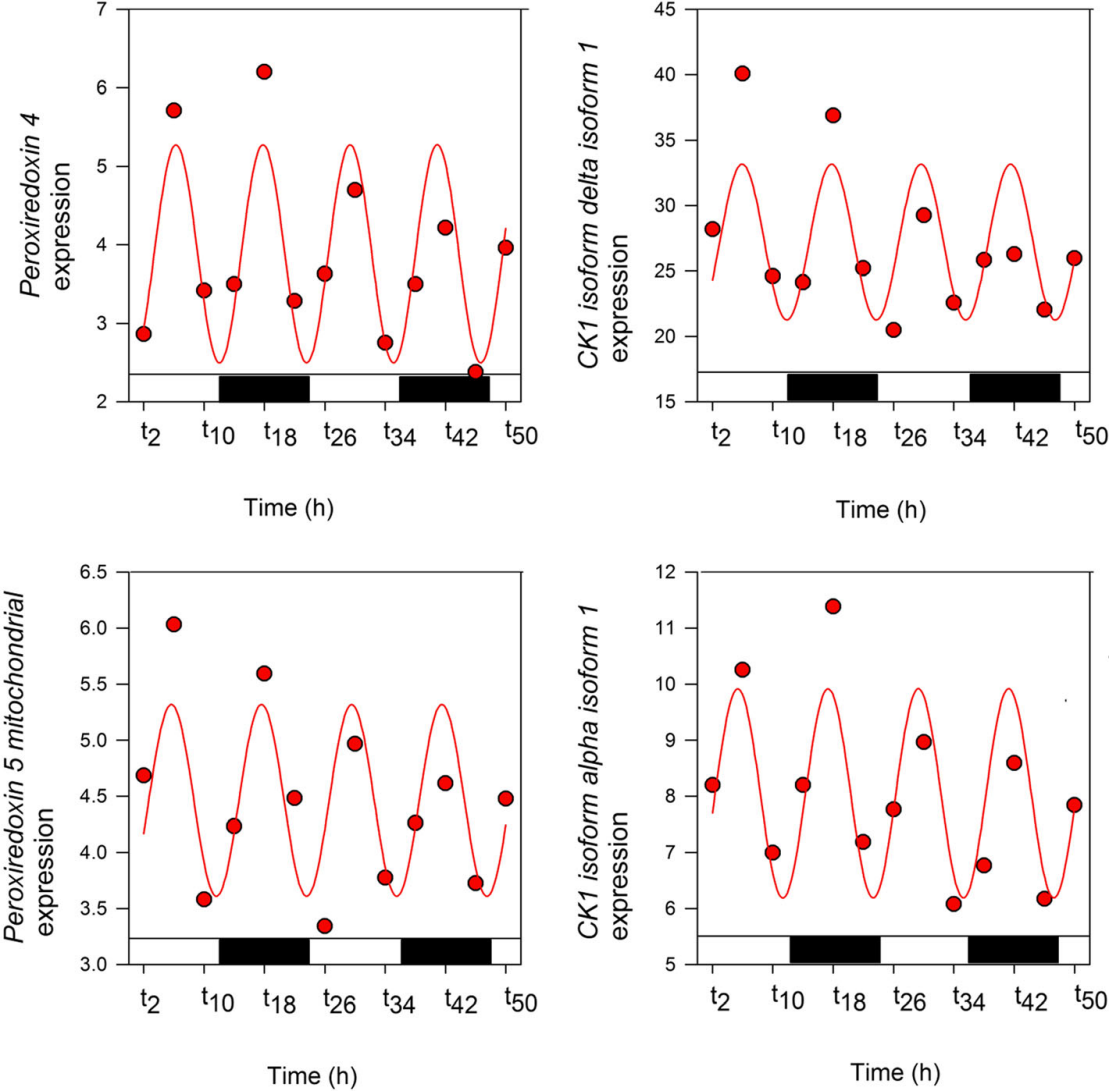
Transcriptome data. Temporal expression patterns of selected *peroxiredoxin* and CK1 genes during 2 days of LD (12:12) for aposymbiotic *Aiptasia* with a 12-h rhythmicity. Fitting curve is based on sinusoidal function

Importantly, the presence of *Symbiodinium* shifted the periodicity of expression of several genes (*n* = 227), from 12 h (aposymbiotic morphs) to 24 h (symbiotic morphs). Three of the five most overrepresented canonical pathways in this dataset (eIF2 signalling pathway, regulation of eIF4/p70S6K signalling, and mTOR signalling) are interrelated and involved in the regulation of translation in response to nutrition or stress (Fig. 4a). Since the mTOR pathway is a pivotal regulator of cell metabolism [13, 14] that is central to nutritional responses [15] and is also involved in entrainment of the circadian clock [14, 16–21], an involvement in switching between aposymbiotic and symbiotic states is to be expected. Other genes in the A12/S24 set encode metabolic or structural proteins, and examples are shown in Fig. 4b. Several of these genes have putative roles in symbiosis, such as *glutamate ammonia ligase,* which encodes a glutamine synthase and has been implicated in maintaining low free ammonia levels in symbiotic cnidarians [22], and the glycerol transporter-type *aquaporin,* which may function in the export of fixed carbon from the symbiont. Two conserved markers of circadian timekeeping, *peroxiredoxin* (two genes: *peroxiredoxin*-4 precursor and *peroxiredoxin*-5 mitochondrial) and *casein kinase 1*, exhibited a 12-h rhythmicity only in the aposymbiotic morphs, expression in symbiotic morphs being arrhythmic (Fig. 5).

**Fig. 6:**
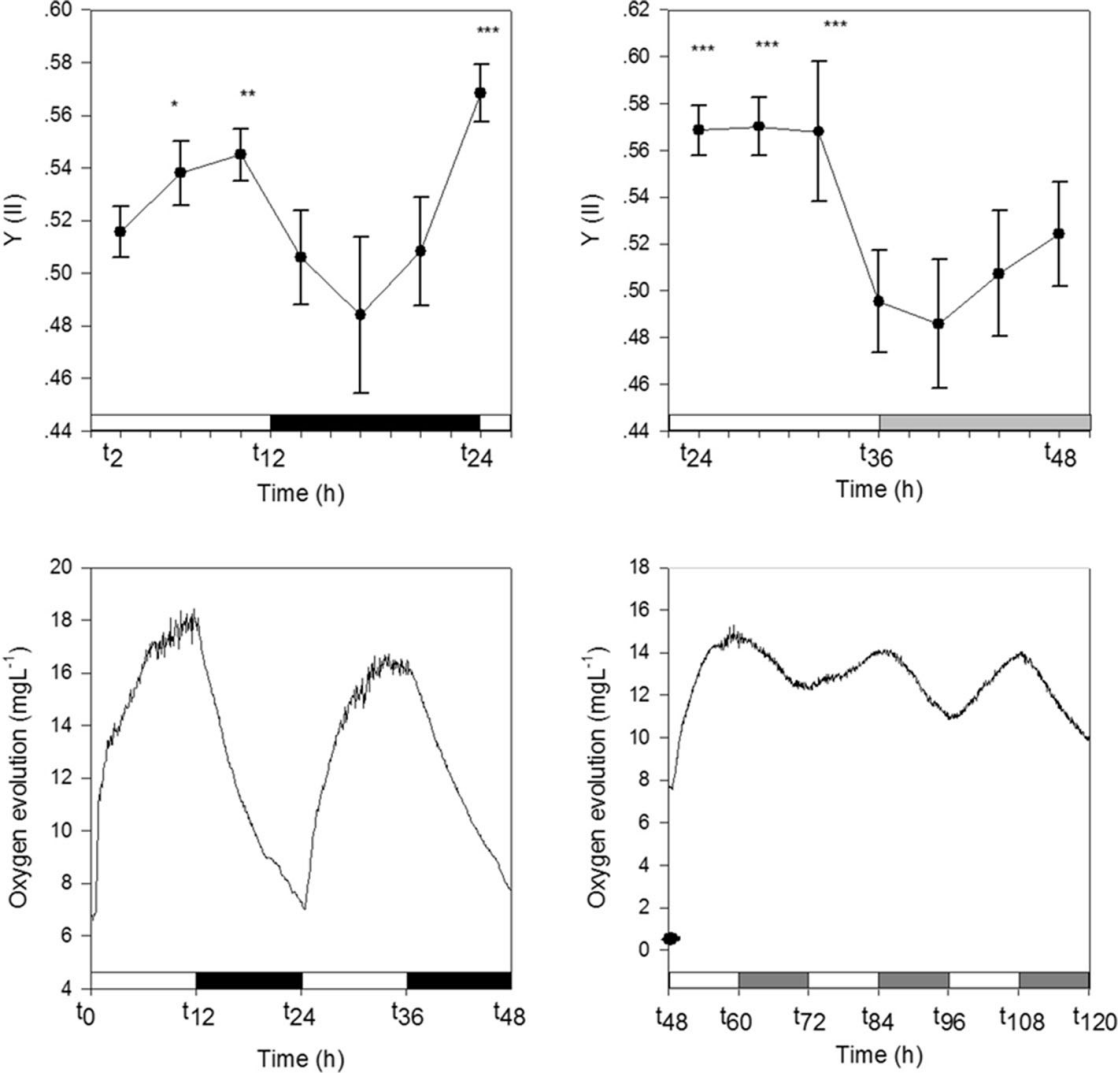
Upper panels—effective PSII quantum yield Y(II) of symbiotic *Aiptasia* (*N* = 4) during 1 LD cycle (12:12) (left panel) followed by 1 cycle under LL (right panel). Lower panels—oxygen evolution from symbiotic *Aiptasia* (*N* = 4) during 2 LD cycles (12:12) (left panel) followed by 3 cycles under LL (right panel). White bars: light period; black bars: dark period; and grey bars: subjective darkness

**Fig. 7:**
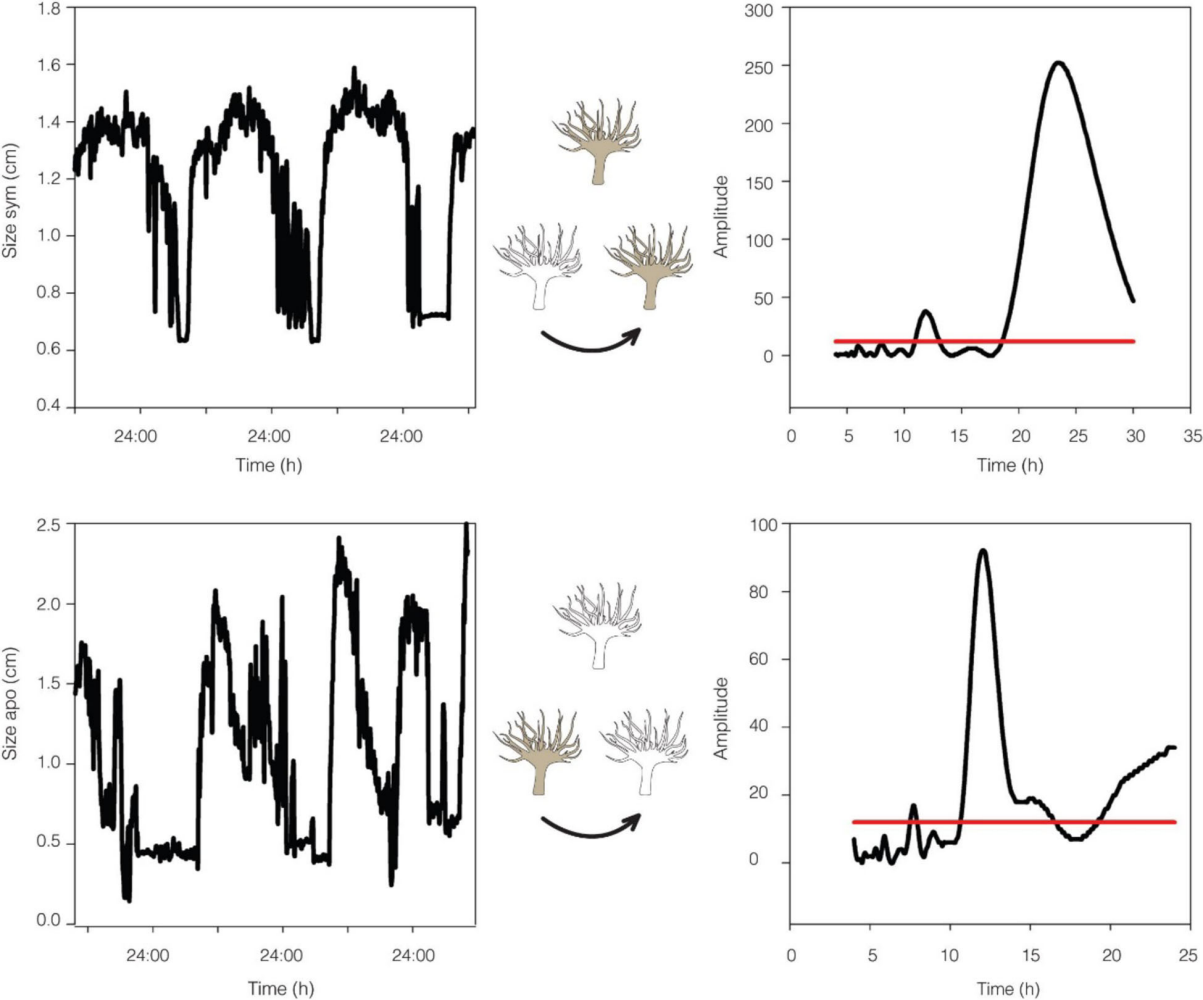
Measurements of *Aiptasia* body expansion and contraction of symbiotic (*N* = 16) and aposymbiotic *Aiptasia* (*N* = 16) during 2 LD cycles (12:12). Left panels show the size of the anemone as detected by the video-tracking system. Right panel shows the periodicity as calculated by LSP software (R. Refinetti). Left panel for symbiotic morphs (upper) revealed a 24.4-h (SD = 1.17) rhythm and aposymbiotic morphs (lower) had a rhythm of 12.42 h (SD = 0.95)

Oxygen evolution measurements revealed a 24-h rhythm of photosynthesis in symbiotic *A. diaphana,* with oxygen evolution maximal at *t*_12_,_36_ (maximum time exposure to light) and minimal at *t*_24_,_48_ (maximum time exposure to dark). A comparable rhythm was observed under constant light (LL) conditions (Fig. 6, lower panel; see the “Methods” section). Similar results were identified by fluorescence measurements using Imaging-PAM. The effective PSII quantum yield Y (II) increased from the 2-h (t_2_) mark, reaching a peak after 10 h (t_10_) of light exposure. These results were confirmed under LL (Fig. 6, upper panel). To investigate the impact of the symbionts on the rhythmic behaviour of *A. diaphana*, the extension and contraction of symbiotic and aposymbiotic individuals (*N* = 16, for each category) was continuously monitored over a cycle ranging between 72 and 96 h. The average periodicity of extension and contraction was 24.11 h (SD ± 1.1) in symbiotic and 12.42 h (SD ± 0.95) in aposymbiotic *A. diaphana* [Fig. 7 right panel as calculated by LSP software (http://www.circadian.org/softwar.html)]. Interestingly, when aposymbiotic *Aiptasia* morphs were re-infected with clade B *Symbiodinium* (CCMP 3345), the periodicity of the inoculated anemones increased to 24.4 h (SD ± 1.17). In contrast, bleached symbiotic sea anemones, thus (aposymbiotic), i.e. without algae, returned to a 12.22-h cycle (SD ± 0.73). The tentacles of symbiotic *A. diaphana* remained open most of the time and contracted only for one period of 6 ± 1.25 h during the dark phase around *t*_18–24_. In contrast, the tentacles of aposymbiotic *A. diaphana*, contracted twice in 24 h, i.e. every ~ 12 h. Contraction commenced once for 4 ± 2 h during the light phase, around 4 h after the lights turned on, and then again for 4 ± 1.35 h during the darkness, 5–6 h after the lights turned off (Fig. 7).

## Discussion

Our findings indicate the importance of the symbiont in the cnidarian-*Symbiodinium* association where the presence of the symbiont was associated with dramatic changes in rhythmic behaviour and gene expression. The predominantly circatidal (i.e. 12 h) rhythmicity of gene expression seen in aposymbiotic morphs was profoundly affected, and a few genes with a circatidal rhythm in this state displayed an approximately diurnal periodicity in the symbiotic organism. As reported for (symbiotic) corals [23], symbiotic *Aiptasia* morphs displayed a diel rhythmicity of oxygen evolution and PSII effective quantum yield under both light dark (12:12) and constant light conditions (Fig. 6). These findings support the hypothesis that regardless of external light, it is the endogenous circadian clock of the symbiont that controls photosynthesis and influences host rhythmicity via metabolite transfer [24, 25] and alterations to the redox state [3]. We have previously shown that cycles of tissue oxygenation driven by the photosymbionts result in diel expression of host genes involved in glycolysis [3], but there are few other precedents for the gene expression patterns of a host being influenced by the presence of symbiotic algae. The unicellular ciliate, *Paramecium bursaria*, contains several hundred green algae (zoochlorellae) that determine the mating reactivity rhythms of the host, probably via the production of photosynthetic products such as maltose and oxygen [26]. Photosynthetic products of symbiotic *Chlorella* can also rescue arrhythmic mutants of white cells (cells with no symbiotic algae) [27]. Additional support for the ability of a symbiont to influence the behaviour of a host is seen in the association between the Hawaiian squid *E. scolopes* and the luminous symbiont *Vibrio fischer*. This symbiosis provides evidence that the presentation of bacterial products is required for cyclic expression of a cryptochrome gene in the symbiotic organ [28]. As previously reported for other cnidarians [11, 12], our results showed a clear diel rhythm in both types of *Aiptasia* morphs for a number of putative endogenous clock genes (Fig. 3b). We therefore suggest that, unlike the squid-vibrio symbiosis, the symbiotic algae in cnidarians do not influence the rhythmicity of core circadian clock components. As might be expected of an inhabitant of coastal environments, the tides have a strong influence, and there are more genes under circatidal regulation (*n* = 5142) in aposymbiotic *A. diaphana* morphs than those (*N* = 1178) cycling on a circadian basis. Whilst the changes in oxygen associated with symbiont photosynthesis might be expected to affect the temporal patterns of gene expression, it is not trivial to explain how a significant number (*n* = 227) of genes are shifted from 12- to 24-h cycling by the presence of the symbiont [with a smaller number (*n* = 16) of genes shifting in the opposite direction]. Clues to the solution come from the temporal patterns of casein kinase 1 (CK1) and peroxiredoxin (PRX) observed here (Fig. 5). Both these genes are circadian markers in mammals and *Drosophila*, and circadian rhythms in mammals can be extended by inhibition of CK1 activity [29]. In contrast, PRX expression displays a circatidal periodicity in *Eurydice* [2], and both circatidal and circadian rhythms are altered by CK1 inhibition [1], suggesting that CK1 may be a component of both clock types. In the case of *A. diaphana*, activity essentially follows a tidal rhythm in aposymbiotic morphs but in symbiotic morphs the rhythm is diel. Both CK1 and PRX oscillate with a circatidal periodicity in aposymbiotic morphs but, surprisingly, are asynchronous in the predominantly diel synchronized symbiotic anemones (data not shown). This suggests that these genes may be key modulators of the circatidal pacemaker.

Several classic HIF-target genes (those encoding the glycolytic enzymes aldolase (Fig. 4b), phosphoglycerate kinase and phosphoglycerate dehydrogenase; Additional file 1: Table S1) were upregulated at night in symbiotic morphs of *A. diaphana.* This observation was also reported for the coral *Acropora* [3], presumably as a consequence of tissue hypoxia driven by respiration. In coral, many stress-response genes are “hard-wired” to the circadian clock [3] and continue to cycle with 24-h periodicity even in the dark. We found no evidence for a similar link in *A. diaphana*. Several of the reported *Acropora* stress-response genes (e.g. calreticulin, protein disulphide isomerase) do not cycle in aposymbiotic *Aiptasia* morphs, but do cycle (with 24-h periodicity) in symbiotic morphs. The implication is that in corals, which have an obligate relationship with *Symbiodinium*, selection pressure has resulted in the direct regulation of certain stress response genes by the circadian clock. This may not have occurred in the sea anemone where the association with the symbiont is facultative. The 24-h photosynthetic rhythms in the algae may also be responsible for synchronising the expression of other physiological genes in the host, such as those involved in ammonium assimilation and transport of fixed carbon.

In behavioural experiments, symbiotic *A. diaphana* displayed diel (24 h) rhythmicity in body and tentacle contraction under the light/dark cycles, whereas aposymbiotic morphs showed approximately 12-h (circatidal) rhythmicity (Fig. 7). The proof of concept reinfection experiment described here where aposymbionts sea anemones were reintroduced represents an important step in understanding the hierarchy of endogenous clocks in symbiotic associations. The observation that aposymbiotic *Aiptasia* morphs returned to a 24-h behavioural rhythm after repopulation with algae clearly demonstrates the importance of the endosymbiotic algae in determining the timing and the duration of the extension and contraction of the body and tentacles, since all other variables (food, flow) were controlled. Both circadian [30–32] and circatidal [33, 34] rhythms of activity have been documented in cnidarians; however, previous studies have not controlled for the presence of the algal symbionts, presumably due to the difficulty in culturing most symbiotic cnidarians without their associated zooxanthellae.

## Conclusion

The work described here is consistent with the hypothesis that symbiotic cnidarians such as *Aiptasia* possess two rhythms entrained by different cues (tidal and solar). Whilst the basal rhythmicity of aposymbiotic morphs presumably reflects evolutionary adaptation to tidal cycles, photosynthetic activity appears to be the primary determinant of host biological pace in symbiotic morphs. The analyses presented here illustrate the complexity of biological rhythms in facultative symbiotic associations such as *Aiptasia*. Given the extent to which the endosymbiont apparently overrides the endogenous rhythms in *Aiptasia*, it remains to be seen whether circatidal rhythms can be detected in *Acropora* or other cnidarians, which have an obligate rather than a facultative association with *Symbiodinium*.

## Methods

Specimens of *Aiptasia diaphana* were collected from Michmoret, a rocky Mediterranean Sea beach in Israel, and maintained in laboratory tanks with artificial seawater under a light/dark cycle (12:12) with illumination of 70 μmol quanta m^−2^ s^−1^ controlled by a Lumen Aqua LED lighting system (Ocean Lights, USA). The water temperature was set to 24 °C. All animals were fed twice a week with newly hatched brine shrimp, and seawater was changed twice per week. To identify the clade present, DNA was extracted from symbiotic *A. diaphana* using the DNeasy kit (Qiagene, Germany) and analysed by RT-PCR, using a set of four clade-specific primers (A-D) following published protocols [35]. The clade was identified as clade B. To remove the *Symbiodinium*, symbiotic *A. diaphana,* grown in a plastic tank, were repeatedly subjected to cold shock treatments (4 °C for 4 h) followed by several days of incubation at 33 °C in the dark. After 7 weeks of such treatments, animals appeared white and the absence of algae was verified by examination under the Imaging PAM and fluorescent stereomicroscope. Aposymbiotic animals were subsequently maintained in filtered (0.45 μm) seawater and fed under the same regime as the symbiotic animals. Using these procedures, animals could be maintained in the laboratory in the bleached state for more than 4 months. For the reverse procedure, cultured *Symbiodinium* clade B (3345 CCMP) were introduced to aposymbiotic *A. diaphana* twice a week and the integration of the algae was monitored under the Imaging PAM. At *t*_6_ (6 h after dark), the average values of effective PSII quantum yield (Y(II), 0.53 ± 0.04, *N* = 16,) were like those of the naturally symbiotic *Aiptasia* (0.52 ± 0.08, *N* = 16) at the same time point. Aposymbiotic *A. diaphana* showed values of 0.00 of effective PSII quantum yield (Y(II)).

Oxygen evolution from symbiotic *A. diaphana* was continuously monitored at 5-min intervals using a 4-channel oxygen meter (OXY4) equipped with four optical oxygen sensors (OPTODs; PreSens, Germany) connected to four dipping probes (DP-PSts). Each chamber contained four individual organisms. Oxygen evolution was monitored over 2 days of (12:12) LD cycle (70 μmol quanta m^−2^ s^−1^) followed by 3 days of constant light (LL) using a constant temperature of 24 °C. Effective quantum yield was measured for symbiotic *A. diaphana* during 1 day of LD (12:12) followed by 1 day LL under the same light intensity and temperature conditions. Four individual animals were analysed every 4 h by Imaging-PAM (pulse amplitude modulation; Maxi-PAM, Walz Gmbh, Effeltrich, Germany), and the resulting images were analysed for each individual with the Imaging-Win software programme (v2.00 m, Walz Gmbh, Effeltrich, Germany). We chose to calculate Effective PSII quantum yield Y(II) values without dark adaptation to avoid unnecessary interruption to the photosynthetic rhythm.

For deep sequencing analyses, symbiotic (sym) and aposymbiotic (apo) animals (total of 104 individuals) were maintained at 24 °C in separate 50-ml Falcon tubes under 12:12 light/dark with illumination of 70 μmol quanta m^−2^ s^−1^. At 4-h intervals (2 h after lights on, 6 h under light, 10 h under light, 2 h after lights off, 6 h in the dark and 10 h in the dark), four apo and four sym individuals (Fig. 1, upper panel) were sampled for RNA analysis by snap freezing in liquid nitrogen and were immediately transferred to − 80 °C for storage. RNA was prepared using a combined RNA extraction procedure including Trizol reagent and an RNeasy Mini Kit (Qiagen) [36]. RNA concentrations were determined using a NanoDrop (ND-1000) spectrophotometer, and the integrity was assessed by microcapillary electrophoresis, using an RNA 6000 Nano LabChip Kit (Agilent 2100 Bioanalyzer, Agilent Technologies, Australia). RNA samples with integrity values (RINs) > 9 were used for deep sequencing analyses. RNAseq libraries were prepared from 1.5-μg aliquots of RNA (*N* = 3–4) for each treatment and time point (total of 13 time points), using the Illumina TruSeq RNA Library Preparation Kit v2 kit, according to the manufacturer’s protocol. Libraries were sequenced by the Technion Genome Centre, Israel Institute of Technology, Haifa, Israel, on the HiSeq 2500 platform with v3 reagents. Analyses of the 100 bp paired-end (PE) RNA-Seq data were carried out at the Bioinformatics Core Facility, National Institute for Biotechnology in the Negev, Ben-Gurion University of the Negev and the Bioinformatics unit of the Mina & Everard Goodman Faculty of Life Sciences, Bar Ilan University, Ramat Gan, Israel. In order to filter out *Symbiodinium* sequence reads, the raw reads were successively aligned to *Aiptasia* and *Symbiodinium* sequences which were publicly available at the time of the study (November 2014). The filtering strategy was intended to retain the maximum number of presumably cnidarian reads, whilst eliminating only reads that were aligned to a known *Symbiodinium* sequence and not aligned to any available cnidarian sequence. For the alignment, we used the SNAP programme, which allows more sequence differences compared to, e.g. BWA-mem or Bowtie, but on the other hand is much faster than BLAST [37]. The following *Symbiodinium* sequence datasets were downloaded: *Symbiodinium minutum* whole genome shotgun sequence from NCBI (GenBank Accession GCA_000507305.1); *Symbiodinium minutum* transcriptome, assembled genome and genome-predicted transcripts from OIST Marine Genomics Unit (Project ID 21 [38] at http://marinegenomics.oist.jp/); and all *Symbiodinium* (tax ID 2949) nucleotide and EST sequences from Genbank. The following cnidarian sequence datasets were downloaded: *Pseudodiploria strigose* (GenBank assembly accession GCA_000751095.1), *Acropora digitifera* (GenBank assembly accession GCA_000222465.1), *Alatina moseri* (GenBank assembly accession GCA_000260875.1), and *Nematostella vectensis* (RefSeq assembly accession GCF_000209225.1) whole genome shotgun sequences from NCBI; The aposymbiotic CC7 *Aiptasia* transcriptome from Pringlelab:(http://pringlelab.stanford.edu/project%20files/AposymbioticAiptasiaTranscriptomeGoodLociForMapping.fa.gz). The downloaded FASTA files of each taxon (*Symbiodinium* or cnidarian) were concatenated and subsequently indexed to allow efficient SNAP search against them. The reads of each sample were aligned to the *Symbiodinium* sequences using SNAP 1.0beta.10 version (http://snap.cs.berkeley.edu/) with the following parameters: -s 50350 -fs -d 20 -f -= --hp. Unmapped reads (average 95% of all reads per sample) were retained as presumed “Aiptasia-originated” reads. In order to save genuine *Aiptasia* reads that may have been mapped to *Symbiodinium* due to the existence of short orthologous sites, all reads that were mapped to *Symbiodinium* were subsequently aligned to the cnidarian sequences with the parameters -s 50350 -fs -d 20 -f -= --hp. Mapped reads (69 ± 17% per sample) were retained and added to the “Aiptasia-originated reads”. We were aware to the possibility that some of these reads aligned to symbiont genes erroneously included in the cnidarian sequences; however, as stated above, we preferred to retain as many presumable cnidarian reads as possible, whilst compromising for minor symbiont contamination. (Contigs formed from retained *Symbiodinium* reads can be detected later using BLAST.) The *Aiptasia*-originated reads from all samples were quality trimmed with Trimmomatic (parameters: LEADING:5 TRAILING:5 MINLEN:36), merged, and a de novo assembled using Trinity [39]. After excluding reads of < 200 bp, the resulting assembly contained 359,881 transcripts and is hereafter designated as the “*Aiptasia* transcriptome”. Profile expression reads from each sample were aligned to the “*Aiptasia* transcriptome” using Bowtie, and the numbers of mapped reads per transcript per sample were quantified using RSEM. Normalized expression values were calculated using two independent methods: TMM-normalized FPKM expression values were generated by RSEM, and transcripts with a normalized value larger than 3 in at least three samples were retained, yielding a total of 25,421 transcripts. The raw counts were normalized using DESeq, and transcripts with normalized value larger than 30 in at least three samples were retained, yielding a total of 29,024 transcripts. Data are available in http://www.ncbi.nlm.nih.gov/sra/SRP073280.

Combining the two transcript sets resulted in 35,492 transcripts, fulfilling either of the above criteria, and this reference was used for further analyses. Functional predictions for this dataset were carried out by BLASTX searching against both the UniProt and UniRef50 databases (UniProt Consortium, 2014). The best hit per transcript with *e* value < 10^−4^ was selected, and the description, Gene ontology, KEGG pathway and conserved domain(s) for each hit retrieved from the corresponding databases. A total of 44,287 proteins were predicted from the 35,492 contigs dataset by the TransDecoder programme [36]. All predicted proteins were reciprocal BLASTP compared against the published *Aiptasia* predicted proteome (29,269 proteins; according to Baumgarten et al. [5]), alignments in relation to NCBI *Homo sapiens* proteins database (Annotation Release 107), with *e* value < 5 × 10^−5^.

Rhythmicity analyses of the temporal data for the transcripts was carried out using the JTK_CYCLE software (v2.1) [7–9], which uses non-parametric algorithms. Tests were carried out separately for the normalized expression values (*p* value cutoff of 0.05) for each *Aiptasia* morph. The expression, description and annotation of these genes are presented in Additional file 1: Table S1. Venn diagrams (Fig. 2) and heat maps (Fig. 1) were generated using Partek® Genomics Suite® software, version 6.6 Copyright ©; 2015 Partek Inc., St. Louis, MO, USA. Pathway analysis of annotated sequences was performed using the IPA software (http://www.ingenuity.com).

Cycling patterns inferred from transcriptomic data were validated by RT-PCR using cDNAs synthesized from 1-μg aliquots of total RNAs that had been extracted with the Verso cDNA synthesis kit (Thermo scientific). Primers were designed for a set of four genes using Primer Quest software (Integrated DNA Technologies) (see Additional file 2: Figure S1). For both symbiotic and aposymbiotic morphs, two-time points (*t*_6_ (6 h under light) and *t*_18_ (6 h under dark)) were selected for each gene. RT PCR reactions were performed using the Corbett RG6000 real-time detection system with GoTaq RT-PCR Master Mix. Each 10 μl contained 5 μl of GoTaq, 0.25 μl each of 10 mM forward and reverse primers, 1.5 μl of distilled deionized water and 3 μl of diluted cDNA (1: 100). The following thermal profile was used: 94 °C for 7 min followed by 45 cycles of 94 °C for 7 s, 60 °C for 15 s and 72 °C for 20 s. Runs were analysed using the ROTOR-GENE6000 series software 1.7, applying a fluorescence threshold of 0.02 in all cases. Data were normalized against the housekeeping gene *RPL11* (encoding ribosomal protein L11), which showed the most stable expression pattern in our experiments, as reported [40]. The relative expression of each sample and gene was calculated using the method of 2^−ΔΔCT^.

Rhythmic expansion and contraction of the anemone body was monitored over a period ranging between 72 and 96 h using video tracking of 16 individual symbiotic and aposymbiotic morphs. This was achieved by dividing the plastic container into eight arenas, with one individual in each. Individual anemones were illuminated with infrared light (to enable continuous camera detection) and with 70 μmol quanta m^−2^ s^−1^ white light (to maintain a light/dark cycle of 12:12), under a constant temperature of 24 °C. The size of each *Aiptasia* was recorded using the EthoVision XT10 software (Noldus Information Technology), and the detection threshold was set to detect moving black pixels. Since this is the first time that the Ethovision tracking system has been used for cnidarian, it was necessary to first calibrate key system parameters. The software parameters for detection were set as follows: grey scaling, dark contrast range between 90 and 150 (depends on apo/sym morph), subject size range 0–125,000 pixels, subject contour erosion turned off, counter dilatation set at 1–5 (depends on apo/sym morphs), video sample rate of 0.0025 frames per second, and no pixel smoothing. The total area measured for each *A. diaphana* individual was calculated using Microsoft Excel. Two independent assays were performed for either symbiotic or asymbiotic morphs, and experiments were repeated after the symbionts had been bleached or the aposymbionts inoculatedreinoculated. To define the rhythmicity of the extension construction behaviour, statistical analyses were performed using LSP software (written by R. Refinetti; http://www.circadian.org/softwar.html), which employs a Lomb-Scargle-based algorithm and is therefore most appropriate for characterization of circadian and circatidal rhythms [41]. For statistical analyses, one-way ANOVA followed by Tukey HSD were used to assess the differences between the experimental treatments regarding periodicity of body expansion and contraction behaviour between symbiotic and aposymbiotic *Aiptasia* and effective PSII quantum yield Y(II) between day and night time. All statistical analyses were conducted using R software, and the results were considered to be statistically significant at *P* < 0.05.

## Additional files

Additional file 1: Expression, description and annotation of genes presnted and anlysed. (XLSX 9383 kb)

Additional file 2: RT-PCR validation of the transcriptome analysis, script data. (DOCX 133 kb)
