# Supplementary material for: Setting the pace: host rhythmic behaviour and gene expression patterns in the facultatively symbiotic cnidarian Aiptasia are determined largely by Symbiodinium
Source: Microbiome. 2018 May 9;6:83. doi: 10.1186/s40168-018-0465-9 (PMC5941691; doi:10.1186/s40168-018-0465-9)
Supplement: Supplementary file 2 — RT-PCR validation of the transcriptome analysis, script data. (DOCX 133 kb) [file 40168_2018_465_MOESM2_ESM.docx]

**Figure and supplement materials**

**RT PCR primers and amplicon sizes.**

**Figure S1** –Correlation of gene expression Log2 of values of 5 genes obtained from RNA-seq analysis and log2 of the expression values obtained using RT-PCR. The analysis takes into consideration two time points for each gene (t_6_ (6 hours under light) and t_18_ (6 hours under dark)) for symbiotic and aposymbiotic *Aiptasia* morphs. Gene expression measured by RT-PCR and RNA-seq was closely correlated (R^2^ = 0.926).

| **Name** | **Amplicon**  **size (bp)** | | **Forward primer** | **Reverse primer** |
| --- | --- | --- | --- | --- |
| ***Cry1*** | | 106 | GATCCTCCATTGCTCGAATAG | GCTTGAGGAATTAGACGGTAG |
| ***Cry2*** | | 97 | CAGGCATCCATATCTCAAGTA | GGCTGTAGAGAGGAACTTTG |
| ***Cry dash*** | | 118 | CCTGTACTTCCTACATGGATTG | CGACTGTGTGGTGGTTAAATA |
| ***Clock*** | | 121 | CAAGGGCTGTGTTAGGTTAG | GTGCTTCTCTTGTGTTGGA |
| ***Rpl11*** | | 125 | AGCCAAGGTCTTGGAGCAGCTTA | TTGGGCCTCTGACAGTACAGTGAACA |


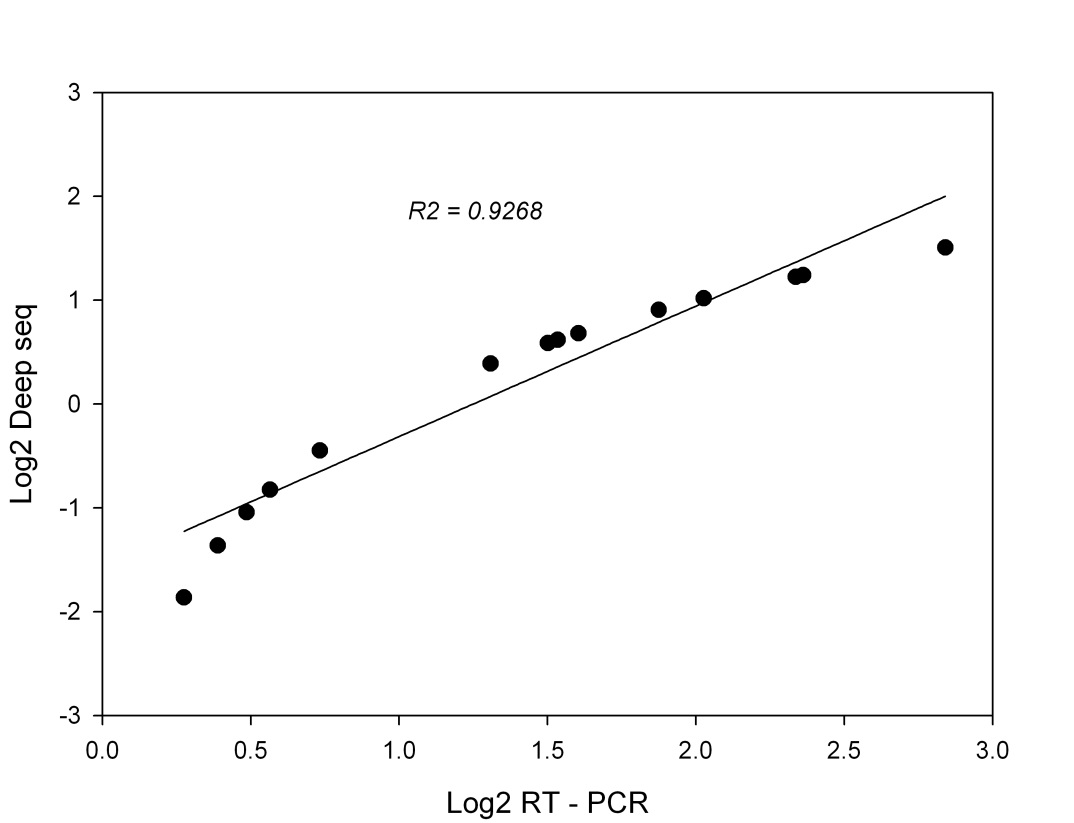


Figure S1

**Computing work flow**

# Outline of bioinformatical analysis Performed for the following publication:

# Setting the pace: Host rhythmic behaviour and gene expression patterns in the facultatively symbiotic cnidarian Aiptasia are determined largely by Symbiodinium.

# Authors: Michal Soreka, ,Yisrael Schnytzera, Hiba Waldman Ben-Ashera, Vered Chalifa Caspib, Chii-Shiarng Chenc, David J. MillerD*, Oren Levya,*

#Analysis was carried out on November 2014

# Author: Menachem Sklarz

# Affiliation: Bioinformatics Core Facility, National institute of Biotechnology in the Negev, Ben-Gurion University of the Negev.

#############################################################

## Step 1: Downloading and concatenating Cnidarian sequences

# a. Make dir for sequences:

cd project_dir

mkdir -p databases/Cnidaria

# b. Get the sequences with the following commands:

wget -O databases/Cnidaria/Nematostella.fa.gz "http://www.ncbi.nlm.nih.gov/Traces/wgs/?download=ABAV01.1.fsa_nt.gz"

wget -O databases/Cnidaria/Pseudodiploria.fa.gz "http://www.ncbi.nlm.nih.gov/Traces/wgs/?download=CCMS01.1.fsa_nt.gz"

wget -O databases/Cnidaria/Acropora.fa.gz "http://www.ncbi.nlm.nih.gov/Traces/wgs/?download=BACK01.1.fsa_nt.gz"

wget -O databases/Cnidaria/Alatina.1.fsa_nt.fa.gz "http://www.ncbi.nlm.nih.gov/Traces/wgs/?download=AHZO01.1.fsa_nt.gz"

wget -O databases/Cnidaria/Alatina.2.fsa_nt.fa.gz "http://www.ncbi.nlm.nih.gov/Traces/wgs/?download=AHZO01.2.fsa_nt.gz"

# c. Get the Aipthasia transcriptome (http://pringlelab.stanford.edu/projects.html)

wget -O databases/Cnidaria/aiptasia/AposymbioticAiptasiaTranscriptomeGoodLociForMapping.fa.gz http://pringlelab.stanford.edu/project%20files/AposymbioticAiptasiaTranscriptomeGoodLociForMapping.fa.gz

# d. The files were unzipped and concatenated:

gzip -cd databases/Cnidaria/Nematostella.fa.gz \

databases/Cnidaria/Pseudodiploria.fa.gz \

databases/Cnidaria/Acropora.fa.gz \

databases/Cnidaria/Alatina.1.fsa_nt.fa.gz \

databases/Cnidaria/Alatina.2.fsa_nt.fa.gz \

databases/Cnidaria/aiptasia/AposymbioticAiptasiaTranscriptomeGoodLociForMapping.fa.gz \

> databases/Cnidaria/Cnidaria_genomes.Apth_trans.fa

# e. Remove all old files

rm -f databases/Cnidaria/Nematostella.fa.gz \

databases/Cnidaria/Pseudodiploria.fa.gz \

databases/Cnidaria/Acropora.fa.gz \

databases/Cnidaria/Alatina.1.fsa_nt.fa.gz \

databases/Cnidaria/Alatina.2.fsa_nt.fa.gz \

databases/Cnidaria/aiptasia/AposymbioticAiptasiaTranscriptomeGoodLociForMapping.fa.gz

# f. The sequences were indexed with snap as prepartion for snap searching of the reads against the downloaded fasta file

snap index \

databases/Cnidaria/Cnidaria_genomes.Apth_trans.fa \

databases/Cnidaria/Cnidaria_genomes.Apth_trans.index \

-bSpace \

-O500 \

-s 25 \

-keysize 5 \

-exact \

-locationSize 5

#############################################################

## Step 2: Downloading and concatenating symbiodinium sequences:

# a. Download and unzip the genome of symbiodinium minutum:

wget -O databases/Symbiodinium/BASF01.1.fa.gz "http://www.ncbi.nlm.nih.gov/Traces/wgs/?download=BASF01.1.fsa_nt.gz"

wget -O databases/Symbiodinium/BASF01.2.fa.gz "http://www.ncbi.nlm.nih.gov/Traces/wgs/?download=BASF01.2.fsa_nt.gz"

gunzip databases/Symbiodinium/BASF01.1.fa.gz

gunzip databases/Symbiodinium/BASF01.2.fa.gz

# b. Download and unzip symbiodinium minutum transcriptome:

wget -O databases/Symbiodinium/Symbiodinium_Minutum_trinity_transcriptome.fa.gz "http://marinegenomics.oist.jp/genomes/download/symbB1_v1.0.transcriptome_trinity.fa.gz"

gunzip databases/Symbiodinium/Symbiodinium_Minutum_trinity_transcriptome.fa.gz

# c. Download and unzip Symbiodinium minutum assembled genome:

wget -O databases/Symbiodinium/Symbiodinium_Minutum_assembled_genome.fa.gz "http://marinegenomics.oist.jp/genomes/download/symbB.v1.0.genome.fa.gz"

gunzip databases/Symbiodinium/Symbiodinium_Minutum_assembled_genome.fa.gz

# d. Download and unzip Predicted Trascripts for Genome Assembly:

wget -O databases/Symbiodinium/Symbiodinium_Minutum_predicted_RNA.fa.gz "http://marinegenomics.oist.jp/genomes/download/symbB.v1.2.augustus.mrna.fa.gz"

gunzip databases/Symbiodinium/Symbiodinium_Minutum_predicted_RNA.fa.gz

# e. Download Symbiodinium nucl database:

esearch -db nuccore -query "txid2949[Organism]" | efetch -format fasta > databases/Symbiodinium/Symbiodinium_NUCL.fasta

# f. Download Symbiodinium EST database:

esearch -db nucest -query "txid2949[Organism]" | efetch -format fasta > databases/Symbiodinium/Symbiodinium_EST.fasta

# g. concatenate the files:

cat databases/Symbiodinium/BASF01.1.fa \

databases/Symbiodinium/BASF01.2.fa \

databases/Symbiodinium/Symbiodinium_Minutum_predicted_RNA.fa \

databases/Symbiodinium/Symbiodinium_Minutum_trinity_transcriptome.fa \

databases/Symbiodinium/Symbiodinium_Minutum_assembled_genome.fa \

databases/Symbiodinium/Symbiodinium_EST.fasta \

databases/Symbiodinium/Symbiodinium_NUCL.fasta > databases/Symbiodinium/Symbiodinium_combined.fa

# h. Remove all old files

rm -f databases/Symbiodinium/BASF01.1.fa \

databases/Symbiodinium/BASF01.2.fa \

databases/Symbiodinium/Symbiodinimu_EST.fasta \

databases/Symbiodinium/Symbiodinium_Minutum_predicted_RNA.fa \

databases/Symbiodinium/Symbiodinium_Minutum_trinity_transcriptome.fa \

databases/Symbiodinium/Symbiodinium_Minutum_assembled_genome.fa \

databases/Symbiodinium/Symbiodinium_NUCL.fasta

# i. The sequences were indexed with snap as prepartion for snap searching of the reads against the downloaded fasta file

snap index \

databases/Symbiodinium/Symbiodinium_combined.fa \

databases/Symbiodinium/Symbiodinium_combined.index \

-bSpace \

-O500 \

-s 25 \

-keysize 5 \

-exact \

-locationSize 5

#############################################################

# Step 3: The reads of each sample were aligned to the Symbiodinium sequences using SNAP 1.0beta.10 version

# a. Reads are aligned to the Symbiodinium sequences with SNAP.

# Note: The command is truncated.

snap paired databases/Symbiodinium/Symbiodinium_combined.index \

A1_AGTTCC_L003_R1_001.fastq.gz \

A1_AGTTCC_L003_R2_001.fastq.gz \

-o mapping_all_to_Symb_DB/sam_files/A1_AGTTCC_L003_001.sam \

-s 50 350 -fs -d 20 -f -= --hp , \

paired databases/Symbiodinium/Symbiodinium_combined.index \

A1_AGTTCC_L003_R1_002.fastq.gz \

A1_AGTTCC_L003_R2_002.fastq.gz \

-o mapping_all_to_Symb_DB/sam_files/A1_AGTTCC_L003_002.sam \

-s 50 350 -fs -d 20 -f -= --hp , \

# ... For each pair of fastq files

# COMMENT: All the alignments are performed thus in one command to save the time required for SNAP to load the index.

# b. For each SAM file produced by SNAP, we then extracted the aligned and non aligned reads with an in-house script, filter_sam.pl:

# This is done for each SAM file separately.

# The filter_sam.pl script is attached

# Note: You can use 'samtools' to get the functionality of this script!

filter_sam.pl \

-s mapping_all_to_Symb_DB/sam_files/sam_files/A1_AGTTCC_L003_002.sam \

-f A1_AGTTCC_L003_R1_002.fastq.gz \

-r A1_AGTTCC_L003_R2_002.fastq.gz \

-o mapping_all_to_Symb_DB/mapped_unmapped_reads/ \

-gz

# ... For each SAM file produced by SNAP.

#############################################################

# Step 4: In order to "rescue" genuine Aiptasia reads that may have mapped to the symbiodinium sequences,

# all reads that were mapped to Symbiodinium were subsequently aligned to cnidarian sequences.

# a. Reads are aligned to the Cnidarian sequences with SNAP.

# Note: The command is truncated.

snap paired databases/Cnidaria/Cnidaria/Cnidaria_genomes.Apth_trans.index \

mapping_all_to_Symb_DB/mapped_unmapped_reads/A1_AGTTCC_L003_R1_001.fastq.gz.mapped.fq.gz \

mapping_all_to_Symb_DB/mapped_unmapped_reads/A1_AGTTCC_L003_R2_001.fastq.gz.mapped.fq.gz \

-o map_mapped_to_cnidaria_DB/sam_files/A1_AGTTCC_L003_001.sam \

-s 50 350 -fs -d 20 -f -= --hp , \

paired databases/Cnidaria/Cnidaria/Cnidaria_genomes.Apth_trans.index \

mapping_all_to_Symb_DB/mapped_unmapped_reads/A1_AGTTCC_L003_R1_002.fastq.gz.mapped.fq.gz \

mapping_all_to_Symb_DB/mapped_unmapped_reads/A1_AGTTCC_L003_R2_002.fastq.gz.mapped.fq.gz \

-o map_mapped_to_cnidaria_DB/sam_files/A1_AGTTCC_L003_002.sam \

-s 50 350 -fs -d 20 -f -= --hp , \

# ... For each pair of fastq files

# COMMENT: All the alignments are performed thus in one command to save the time required for SNAP to load the index.

# b. For each SAM file produced by SNAP, we then extracted the aligned and non aligned reads:

# This is done for each SAM file separately.

filter_sam.pl \

-s map_mapped_to_cnidaria_DB/sam_files/A1_AGTTCC_L003_001.sam \

-f mapping_all_to_Symb_DB/mapped_unmapped_reads/A1_AGTTCC_L003_R1_001.fastq.gz.mapped.fq.gz \

-r mapping_all_to_Symb_DB/mapped_unmapped_reads/A1_AGTTCC_L003_R2_001.fastq.gz.mapped.fq.gz \

-o map_mapped_to_cnidaria_DB/mapped_unmapped_reads \

-gz

# ... For each SAM file produced by SNAP.

#############################################################

# Step 5. All putative "Aiptasia-originated reads" were concatenated into a single file per sample per direction:

# Putative "Aiptasia-originated reads" = reads that did NOT map to Symbiodinium sequences OR DID map to Cnidarian sequences

# a. Concatenating the reads:

gzip -cd \

mapping_all_to_Symb_DB/mapped_unmapped_reads/A1_AGTTCC_L003_R1_001.fastq.gz.unmapped.fq.gz \

mapping_all_to_Symb_DB/mapped_unmapped_reads/A1_AGTTCC_L003_R1_002.fastq.gz.unmapped.fq.gz \

mapping_all_to_Symb_DB/mapped_unmapped_reads/A1_AGTTCC_L003_R1_003.fastq.gz.unmapped.fq.gz \

map_mapped_to_cnidaria_good_DB/mapped_unmapped_reads/A1_AGTTCC_L003_R1_001.fastq.gz.mapped.fq.gz.mapped.fq.gz \

map_mapped_to_cnidaria_good_DB/mapped_unmapped_reads/A1_AGTTCC_L003_R1_002.fastq.gz.mapped.fq.gz.mapped.fq.gz \

map_mapped_to_cnidaria_good_DB/mapped_unmapped_reads/A1_AGTTCC_L003_R1_003.fastq.gz.mapped.fq.gz.mapped.fq.gz \

> reads4assembly/A1.F.merge.fq

# ... for each sample, once for forward reads and once for reverse reads.

# b. Adding Trinity tags (/1 and /2) to the ends of the sequences:

awk '{ if (NR%4==1) { gsub(" ","_"); print $0"/1" } else { print } }' \

reads4assembly/A1.F.merge.fq \

> reads4assembly/A1.F.tags.fq

awk '{ if (NR%4==1) { gsub(" ","_"); print $0"/2" } else { print } }' \

reads4assembly/A1.R.merge.fq \

> reads4assembly/A1.R.tags.fq

# ... For each sample.

#############################################################

# Step 6: De-novo assembly of the transcriptome using Trinity:

# a. Transcriptome assembly with Trinity:

Trinity \

--trimmomatic \

--JM 140G \

--CPU 20 \

--seqType fq \

--grid_conf SGE_Trinity_conf.txt \

--full_cleanup \

--quality_trimming_params "LEADING:5 TRAILING:5 MINLEN:36" \

--min_kmer_cov 2 \

--output Trin_Apthasia \

--left A1.F.tags.fq,A10.F.tags.fq,A11.F.tags.fq,A12.F.tags.fq,A13.F.tags.fq,A2.F.tags.fq,A3.F.tags.fq,A4.F.tags.fq,A5.F.tags.fq,A6.F.tags.fq,A7.F.tags.fq,A8.F.tags.fq,A9.F.tags.fq,S1.F.tags.fq,S10.F.tags.fq,S11.F.tags.fq,S12.F.tags.fq,S13.F.tags.fq,S2.F.tags.fq,S3.F.tags.fq,S4.F.tags.fq,S5.F.tags.fq,S6.F.tags.fq,S7.F.tags.fq,S8.F.tags.fq,S9.F.tags.fq \

--right A1.R.tags.fq,A10.R.tags.fq,A11.R.tags.fq,A12.R.tags.fq,A13.R.tags.fq,A2.R.tags.fq,A3.R.tags.fq,A4.R.tags.fq,A5.R.tags.fq,A6.R.tags.fq,A7.R.tags.fq,A8.R.tags.fq,A9.R.tags.fq,S1.R.tags.fq,S10.R.tags.fq,S11.R.tags.fq,S12.R.tags.fq,S13.R.tags.fq,S2.R.tags.fq,S3.R.tags.fq,S4.R.tags.fq,S5.R.tags.fq,S6.R.tags.fq,S7.R.tags.fq,S8.R.tags.fq,S9.R.tags.fq

# b. Reads from each sample were aligned to the "Aiptasia transcriptome" using Bowtie,

# and the numbers of mapped reads per transcript per sample were quantified using RSEM.

# Both were done with the Trinity utility 'align_and_estimate_abundance.pl'

align_and_estimate_abundance.pl \

--aln_method bowtie \

--est_method RSEM \

--seqType fq \

--trinity_mode \

--transcripts Trin_Apthasia.Trinity.fasta \

--left A1.F.tags.fq \

--right A1.R.tags.fq \

--output_dir trin_map/A1 \

--output_prefix A1

# ... For each sample

# c. TMM-normalized FPKM expression values were generated by RSEM, using the Trinity utility 'abundance_estimates_to_matrix.pl'

/fastspace/bioinfo_apps/trinityrnaseq_r20140717/util/abundance_estimates_to_matrix.pl \

--est_method RSEM \

--out_prefix trin_stats/Apthasia \

A1.isoforms.results \

A2.isoforms.results \

A3.isoforms.results \

A4.isoforms.results \

A5.isoforms.results \

A6.isoforms.results \

A7.isoforms.results \

A8.isoforms.results \

A9.isoforms.results \

A10.isoforms.results \

A11.isoforms.results \

A12.isoforms.results \

A13.isoforms.results \

S1.isoforms.results \

S2.isoforms.results \

S3.isoforms.results \

S4.isoforms.results \

S5.isoforms.results \

S6.isoforms.results \

S7.isoforms.results \

S8.isoforms.results \

S9.isoforms.results \

S10.isoforms.results \

S11.isoforms.results \

S12.isoforms.results \

S13.isoforms.results

#############################################################

# Step 7: Statistical analysis of the counts table:

# a. The raw counts were also normalized using DESeq:

# The following is R code!

library ("DESeq")

require (plyr)

countTable = read.table("Apthasia.counts.matrix",

header = T,

row.names = 1)

countTable.rounded = round(countTable) #convert to integers by rounding, otherwise DESeq will not accept it

design = data.frame(row.names = colnames(countTable),

condition = colnames(countTable),

libType = rep("paired-end", 26))

condition = design$condition

cds = newCountDataSet(countTable.rounded, condition)

cds = estimateSizeFactors(cds)

sizeFactors(cds)

cds.raw = counts(cds)

cds.norm = counts(cds, normalized = T)

write.table(cds.norm, file = "DESeq_norm_counts_matrix.txt", sep = "\t")

# b. "... transcripts with normalized value larger than 30 in at least 3 samples were retained":

# The following is R code!

# contig_length_file is a table with two columns: transcript name and length:

contig_length_file <- "fasta.titles.txt"

# First three lines:

# c5_g1_i1 236

# c11_g1_i1 209

# c18_g1_i1 203

# count_file is the table of raw counts

count_file <- "Apthasia.counts.matrix"

# count_file is the table of DESeq-normalized counts

norm_count_file <- "DESeq_norm_counts_matrix.txt"

# Cutoffs:

# Minimum number of samples expressing the transcript

f_n <- 3

# Minimum normalized value

f_x <- 30

# Minimum length

length_cutoff <- 200

# Read lengths file and add column names

Trin.lengths <- read.table(contig_length_file,he=F)

names(Trin.lengths) <- c("Iso","Length")

# Read counts file

Trin.counts <- read.table(count_file, strings = F, he = F, skip = 1)

# Read column names

cnts_col_names <- read.table(count_file, nrows = 1, stringsAsFactors = F)

# Add 'counts_' to column names

names(Trin.counts) <- c("Iso", paste("counts", cnts_col_names, sep = "_"))

# Read DESeq file

Trin.DESeq <- read.table(norm_count_file, strings = F, he = F, skip = 1)

# Read column names

deseq_col_names <- read.table(norm_count_file, nrows = 1, stringsAsFactors = F)

# Add 'DESeq_' to column names

names(Trin.DESeq) <- c("Iso", paste("DESeq", deseq_col_names, sep = "_"))

# Merge length, counts and normalized counts:

all.info <- merge(Trin.lengths, Trin.counts, sort = F)

all.info <- merge(all.info, Trin.DESeq, sort = F)

# Filter by length

all.info <- all.info[all.info$Length >= length_cutoff, -2]

# Filter by minimum DESeq - at least n smaples with DESeq>X

t1 <- apply(all.info[,28:53], MARGIN = 1, FUN = function(x) sum(x > f_x) >= f_n)

all.info <- all.info[t1,]

# Write files for JTK_CYCLE

write.table(all.info[,c(1,28:40)],"Apthasia.DESeq.A.4JTK.matrix.txt", quote = F, row.names = F, sep = "\t")

write.table(all.info[,c(1,41:53)],"Apthasia.DESeq.S.4JTK.matrix.txt", quote = F, row.names = F, sep = "\t")

write.table(data.frame(Iso=all.info[,1]),"Apthasia.DESeq.AS.4JTK.annot.txt", quote = F, row.names = F)

# c. "... transcripts with a normalized value (TMM) larger than 3 in at least 3 samples were retained":

# The following is R code!

Trin.lengths <- read.table("fasta.titles.txt",he=F)

names(Trin.lengths) <- c("Iso","Length")

# Read counts file

Trin.counts <- read.table("Apthasia.counts.matrix", strings = F, he = F, skip = 1)

col_names <- read.table("Apthasia.counts.matrix", nrows = 1, stringsAsFactors = F)

names(Trin.counts) <- c("Iso",paste("counts", col_names, sep = "_"))

# Read TMM file

Trin.TMM <- read.table("Apthasia.TMM.fpkm.matrix", strings = F, he = F, skip = 1)

col_names <- read.table("Apthasia.TMM.fpkm.matrix", nrows = 1, stringsAsFactors = F)

names(Trin.TMM) <- c("Iso",paste("TMM", col_names, sep = "_"))

all.info <- merge(Trin.lengths, Trin.counts, sort = F)

all.info <- merge(all.info, Trin.TMM, sort = F)

# Filter by length

all.info <- all.info[all.info$Length >= 200, -2]

# Filter by minimum TMM - at least 2 smaples with TMM>X

f_n <- 3

f_x <- 3

t1 <- apply(all.info[,28:53], MARGIN = 1, FUN = function(x) sum(x > f_x) >= f_n)

all.info <- all.info[t1,]

# Write files for JTK_CYCLE

write.table(all.info[,c(1,28:40)],"Apthasia.TMM.A.gt200.maxsig3.4JTK.matrix", quote = F, row.names = F, sep = "\t")

write.table(all.info[,c(1,41:53)],"Apthasia.TMM.S.gt200.maxsig3.4JTK.matrix", quote = F, row.names = F, sep = "\t")

write.table(data.frame(Iso=all.info[,1]),"Apthasia.TMM.AS.gt200.maxsig3.4JTK.annot", quote = F, row.names = F)

# d. "Combining the two transcript sets resulted in 35,492 transcripts, fulfilling either of the above criteria, and this reference was used for further analyses"

# The following is R code!

# Read tables of transcripts that passed filtering and create combined list in Contig_list_TMM_or_DESeq_filtering

DESeq_passed <- read.table("Apthasia.DESeq.AS.4JTK.annot.txt",

header = T)

TMM_passed <- read.table("Apthasia.TMM.AS.gt200.maxsig3.4JTK.annot",

header = T)

Combined_list <- merge(DESeq_passed,

TMM_passed,

all = T)

write.table(data.frame(Iso=Combined_list),

file = "Contig_list_TMM_or_DESeq_filtering.txt",

quote = F,

row.names = F)

# Read full TMM table and extract those that passed filtering. Save to Unified_filtered_list_TMM_signals

Trin_TMM <- read.table("Apthasia.TMM.fpkm.matrix",

stringsAsFactors = F,

header = F,

skip = 1)

Trin_TMM_passed <- merge(x = Trin_TMM,

y = Combined_list,

by.x = "V1",

by.y = "Iso")

write.table(x = Trin_TMM_passed,

file = "Unified_filtered_list_TMM_signals.txt",

quote = F,

row.names = F,

sep = "\t")

# Read full DESeq table and extract those that passed filtering. Save to Unified_filtered_list_DESeq_signals

Trin_DESeq <- read.table("DESeq_norm_counts_matrix.txt",

stringsAsFactors = F,

header = F,

skip = 1)

Trin_DESeq_passed <- merge(Trin_DESeq,

Combined_list,

by.x = "V1",

by.y = "Iso")

write.table(Trin_DESeq_passed,

file = "Unified_filtered_list_DESeq_signals.txt",

quote = F,

row.names = F,

sep = "\t")

# e. "Rhythmicity analyses of the temporal data for the transcripts was carried out using the JTK_CYCLE software (v2.1)":

# The following is R code!

source("JTK_CYCLE.R")

options(stringsAsFactors = FALSE)

# Defined input files

input_dir <- "./"

annot_file <- "Contig_list_TMM_or_DESeq_filtering.txt"

DESeq_file <- "Unified_filtered_list_DESeq_signals.txt"

TMM_file <- "Unified_filtered_list_TMM_signals.txt"

annot_path <- paste(input_dir, annot_file, sep="")

deseq_path <- paste(input_dir, DESeq_file, sep="")

tmm_path <- paste(input_dir, TMM_file, sep="")

# Read files

annot <- read.delim(annot_path)

deseq_data <- read.delim(deseq_path)

tmm_data <- read.delim(tmm_path)

# Define datasets: DESeq and TMM for each of A and S samples:

deseq_data_A <- deseq_data[,1:14]

deseq_data_S <- deseq_data[,c(1, 15:27)]

tmm_data_A <- tmm_data[,1:14]

tmm_data_S <- tmm_data[,c(1, 15:27)]

# Define JTK parameters:

requested_priods <- c(3, 6)

total_nr_time_points <- 13

nr_reps_per_time_point <- 1

# Define function for executing JTK:

run_jtk <- function (annot, data, requested_priods, project_name) {

rownames(data) <- data[,1]

data <- data[,-1]

#jtkdist(ncol(data))

jtkdist(total_nr_time_points, nr_reps_per_time_point)

periods <- requested_priods

jtk.init(periods,4)

cat("JTK analysis started on",date(),"\n")

flush.console()

st <- system.time({

res <- apply(data,1,function(z) {

jtkx(z)

c(JTK.ADJP,JTK.PERIOD,JTK.LAG,JTK.AMP)

})

res <- as.data.frame(t(res))

bhq <- p.adjust(unlist(res[,1]),"BH")

res <- cbind(bhq,res)

colnames(res) <- c("BH.Q","ADJ.P","PER","LAG","AMP")

results <- cbind(annot,res[annot$Iso,],data[annot$Iso,])

results <- results[order(res$ADJ.P,-res$AMP),]

})

print(st)

save(results,file=paste("JTK",project_name,"rda",sep="."))

write.table(results,file=paste("JTK",project_name,"txt",sep="."),row.names=F,col.names=T,quote=F,sep="\t")

}

# Execute JTK on each of the 4 datasets:

run_jtk (annot, deseq_data_A, requested_priods, "DESeq_A")

run_jtk (annot, deseq_data_S, requested_priods, "DESeq_S")

run_jtk (annot, tmm_data_A, requested_priods, "TMM_A")

run_jtk (annot, tmm_data_S, requested_priods, "TMM_S")

script

#!/usr/bin/perl

#$ -N filt_sam

#$ -cwd

#$ -V

#$ -S /usr/bin/perl

use strict;

use Storable;

use Data::Dumper;

use Getopt::Long;

use File::Basename;

#$ -q bioinfo.q@sge214

my $usage = <<__USAGE__;

This program extracts mapped and unmapped reads from SAM files.

The SAM file includes both mapped and unmapped reads. The reads are extracted and written to four files, forward mapped and unmapped reads and reverse mapped and unmapped reads.

Note: You can use samtools to get the functionality of this script:

1. Discard unmapped reads with "samtools view -F 4"

2. Convert to fastq with "samtools fastq"

Parameters:

-----------

-s The SAM file

-f The file from which to read the forward reads.

-r The file from which to read the reverse reads.

-o The directory in which to create the output files.

Advanced paramsters

--------------------

The script can be executed in two stages:

1. filter_sam.pl is executed with <-hash hashname>. A hash index will be stored in hashname.

2. filter_sam.pl is executed with <-divbin hashname>. The files will be creates based on the hash index hashname.

-hash hashname Reads the SAM file, creates a hash and stores it in hashname.

-divbin Create the -f and -f files from the hash created

If -hash is not passed, both steps will be performed in one go.

Usage examples:

----------------

# Make hash:

perl filter_sam.pl -s <sam file> -f <forward> -r <reverse> -hash <bin file for hash> -o <out dir> -gz

# Make mapped and unmapped files based on hash

perl filter_sam.pl -s <sam file> -f <forward> -r <reverse> -divbin <bin file for hash> -o <out dir>

# Do both stages in one go:

perl filter_sam.pl -s <sam file> -f <forward> -r <reverse> -o <out dir>

1. You must specify o, f, r, and s

2. Add the -gz flag if the output files should be gzipped

3. MAKE SURE THEIR ARE NO TRINITY TAGS. THE NAMES IN BOTH PAIRS SHOULD BE IDENTICAL.

4. A pair will be considered mapped ONLY IF both sequences in the pair mapped (not necessarily to the same chromosome)

5. If a sequence in a pair is missing from the sam file, this script will not identify the problem. The forward and reverse sequences from -r and -f will be copied into mapped or unmapped, depending on the state of the single sequence in the sam file.

__USAGE__

;

my $sam;

my $forward;

my $reverse;

my $hashonly;

my $divonly;

my $outdir;

my $gz;

GetOptions ("s=s" => \$sam, # numeric

"f=s" => \$forward, # string

"r=s" => \$reverse,

"o=s" => \$outdir,

"hash=s" => \$hashonly,

"divbin=s" => \$divonly,

"gz" => \$gz) # flag

or die("Error in command line arguments\n");

die "You must define s, o, f and r! $usage\n" if ($sam eq "" or $forward eq "" or $reverse eq "" or $outdir eq "");

die "You can't define both hashonly and divonly!!\n\n$usage\n" if (defined $hashonly and defined $divonly);

$| = 1; # Force immediate flush

die "Dir $outdir does not exist. Create it first!" if (not ( -d "$outdir" ));

# Remove final slash from outdir name, if exists:

$outdir =~ s/(.*)\/$/$1/;

my %seq_hash;

my $count;

# Create hash only if no binary hash specified:

if (not defined($divonly)) {

print STDERR localtime()."\tCreating hash from sam file $sam:\n";

open(IN_SAM,"<",$sam) or die "$!";

print STDERR localtime()."\tReading header:\n";

while (chomp(my $line=<IN_SAM>)) {

last if ($line !~ /^@/); # Read lines till end of header

$count++;

print STDERR "line $count\n" if ($count%100000 == 0);

}

$count=0;

print STDERR localtime()."\tReading sequences:\n";

while (chomp(my $line=<IN_SAM>)) { # Read lines till end of file. Create hash with 1 for match and 0 for mismatch

my @line = split(/\t/,$line);

# If this is the first sequence in a pair -> Set the hash value to 0 if unmapped or 1 if mapped

if (not exists($seq_hash{$line[0]})) {

if ($line[2] eq "*") {

$seq_hash{$line[0]} = 0;

}

else {

$seq_hash{$line[0]} = 1;

}

}

# If this is the second sequence in a pair -> Change the value to 0 if unmapped. Otherwise, leave as for first in pair

# THIS FEATURE WAS NOT TESTED, BECAUSE THERE WERE NO SUCH CASES. CHECK THE SAM FILE WITH check_paired_sam.pl TO CHECK IF THERE ARE SUCH ISSUES.

else {

if ($seq_hash{$line[0]} == 1 and $line[2] eq "*") {

$seq_hash{$line[0]} = 0;

}

}

$count++;

print STDERR "line $count\tSequence ".$line[0]."\n" if ($count%100000 == 0);

}

}

# Storing binary hash if specified file submitted in hashonly argument

if (defined($hashonly)) {

print STDERR localtime()."\tStoring hash to disk\n";

store \%seq_hash, $hashonly;

exit;

}

# If divonly specified, the hash is read in from the binary file

if (defined($divonly)) {

print STDERR localtime()."\tReading hash from $divonly\n";

my $hashref = retrieve($divonly);

%seq_hash = %$hashref;

# print Dumper $hashref;exit;

}

my $count=0;

print STDERR localtime()."\tCreating mapped and unmapped files:\n";

# If input files are compressed, stream from gzipped files

if ($forward=~/\.gz$/) {

open(IN_F,"-|","gzip -cd $forward ") or die "$!";

open(IN_R,"-|","gzip -cd $reverse ") or die "$!";

}

# Otherwise, stream from regular text file

else {

open(IN_F,"<",$forward) or die "$!";

open(IN_R,"<",$reverse) or die "$!";

}

# if gz flag specified, stream to compressed fq files

if (defined($gz)) {

open(OUT_M_F,"|-","gzip -c > $outdir/".basename($forward).".mapped.fq.gz") or die "$!";

open(OUT_UM_F,"|-","gzip -c > $outdir/".basename($forward).".unmapped.fq.gz") or die "$!";

open(OUT_M_R,"|-","gzip -c > $outdir/".basename($reverse).".mapped.fq.gz") or die "$!";

open(OUT_UM_R,"|-","gzip -c > $outdir/".basename($reverse).".unmapped.fq.gz") or die "$!";

}

# otherwise, output to regular files

else {

open(OUT_M_F,">","$outdir/".basename($forward).".mapped.fastq") or die "$!";

open(OUT_UM_F,">","$outdir/".basename($forward).".unmapped.fastq") or die "$!";

open(OUT_M_R,">","$outdir/".basename($reverse).".mapped.fastq") or die "$!";

open(OUT_UM_R,">","$outdir/".basename($reverse).".unmapped.fastq") or die "$!";

}

my ($line1_F,$line2_F,$line3_F,$line4_F,$line1_R,$line2_R,$line3_R,$line4_R);

while (my $line1_F = <IN_F>) {

# Read 4 lines from F and 4 lines from R

$line2_F = <IN_F>;

$line3_F = <IN_F>;

$line4_F = <IN_F>;

$line1_R = <IN_R>;

$line2_R = <IN_R>;

$line3_R = <IN_R>;

$line4_R = <IN_R>;

# Get sequence name, terminating at first whitespace

my ($seq) = $line1_F=~/^@(\S*)\s/;

# If the sequence exists in the hash, write to mapped R and F files

if ($seq_hash{$seq} == 1) {

print OUT_M_F "$line1_F$line2_F$line3_F$line4_F";

print OUT_M_R "$line1_R$line2_R$line3_R$line4_R";

}

# otherwise, write to unmapped R and F files

else {

print OUT_UM_F "$line1_F$line2_F$line3_F$line4_F";

print OUT_UM_R "$line1_R$line2_R$line3_R$line4_R";

}

$count++;

print STDERR "Sequence # $count\t($seq)\n" if ($count%100000 == 0);

}

exit;

# Optional - Remove e and o files

# NOT IMPLEMENTED YET

my $ofile = $ENV{JOB_NAME}.".o".$ENV{JOB_ID};

my $efile = $ENV{JOB_NAME}.".e".$ENV{JOB_ID};

print STDOUT "stdout\n$ofile";

print STDERR "stderr\n$efile";

qx/mv $ofile qsub_temp\//;

qx/mv $efile qsub_temp\//;
